# Perioperative management of antiplatelet treatment in patients undergoing isolated coronary artery bypass grafting in Dutch cardiothoracic centres

**DOI:** 10.1007/s12471-017-1006-z

**Published:** 2017-06-13

**Authors:** P. W. A. Janssen, D. M. F. Claassens, L. M. Willemsen, T. O. Bergmeijer, P. Klein, J. M. ten Berg

**Affiliations:** 1 St. Antonius Center for Platelet Function Research, Nieuwegein, The Netherlands; 2 Department of Cardiology, St. Antonius Hospital, Nieuwegein, The Netherlands; 3 Department of Cardiothoracic Surgery, St. Antonius Hospital, Nieuwegein, The Netherlands

**Keywords:** Antiplatelet treatment, P2Y_12_ inhibitor, CABG, Acute coronary syndrome, Clopidogrel, Acetylsalicylic acid

## Abstract

**Background:**

International guidelines do not provide uniform recommendations regarding the use of antiplatelet treatment in the perioperative period in patients undergoing coronary artery bypass grafting (CABG).

**Methods:**

A questionnaire was sent to all 16 cardiothoracic centres in the Netherlands to determine which antiplatelet treatment is used in the perioperative setting. Furthermore, a single-centre prospective observational cohort study was performed which included all patients undergoing isolated CABG in July 2014.

**Results:**

Eleven centres responded to the survey. Acetylsalicylic acid monotherapy was discontinued before surgery in 6 centres. In patients with an acute coronary syndrome receiving dual antiplatelet therapy (DAPT), most centres discontinued the P2Y_12_ inhibitor preoperatively. DAPT was restarted after surgery in 4 centres. However, 6 centres continued DAPT in patients who had undergone coronary stenting within one month of surgery. In patients with coronary stents, variation in the management of antiplatelet therapy increased in proportion to the interval between stenting and surgery. A total of 70 patients were included in the registry. Acetylsalicylic acid monotherapy was discontinued in 51% of patients and restarted in all patients. P2Y_12_ inhibitor treatment was discontinued before surgery in 70% of patients and re-initiated after CABG in 29%.

**Conclusions:**

Major differences were observed in the preoperative and postoperative management of antiplatelet treatment between different Dutch cardiothoracic centres and within a single centre. Part of this variation is probably due to lack of evidence and differences between the current guidelines; however, many of the strategies were not in accordance with any of these guidelines.

**Electronic supplementary material:**

The online version of this article (doi: 10.1007/s12471-017-1006-z) contains supplementary material, which is available to authorized users.

## Introduction

Most patients scheduled to undergo coronary arterial bypass grafting (CABG) are treated with acetylsalicylic acid (ASA) with or without a P2Y_12_ inhibitor (clopidogrel, prasugrel, ticagrelor) before surgery. The current guidelines from the American College of Cardiology (ACC)/American Heart Association (AHA), European Society of Cardiology (ESC) and European Association for Cardio-Thoracic Surgery (EACTS) provide different recommendations regarding the continuation or (temporary) cessation of antiplatelet drugs during the perioperative period [1–4]. In general, it is recommended to continue ASA during and after CABG [1–4]. However, some guidelines state that it can be reasonable to discontinue ASA several days before CABG in patients with stable coronary heart disease [2, 3]. The guidelines are consistent in their advice to discontinue P2Y_12_ inhibitors before surgery in stable patients without recent coronary stent implantation, although there is no consistency regarding the timing of discontinuation. In high-risk groups, i. e. patients who have recently undergone coronary stent implantation [2] or patients with a high risk for thrombotic events [4], it is recommended not to interrupt dual antiplatelet therapy (DAPT) treatment. Physicians have to decide whether the increased risk of bleeding with continued antiplatelet therapy outweighs the risk of thrombotic events associated with the discontinuation of these drugs before CABG. The use of DAPT after CABG in patients who recently experienced an acute coronary syndrome (ACS) is also a subject of debate [5]. Recent reports have shown that treatment is not re-initiated after surgery in a large portion of patients [6], despite the fact that re-initiation is recommended in the guidelines [3, 4].

We aimed to describe the use of antiplatelet treatment in the perioperative period in patients undergoing CABG in contemporary practice in the Netherlands.

## Materials and methods

First, a survey was sent to all 16 centres in the Netherlands in which CABG surgery is performed. The head of the department of each centre was contacted. The survey consisted of both open and closed questions so that respondents could indicate how predefined groups of patients would be treated in general (i. e. mono antiplatelet therapy versus DAPT, patients after ACS and/or recent stent implantation) and which patients were exempted from standard treatment protocols. The survey is shown online as Electronic Supplementary Material.

Second, we conducted a prospective, observational pilot study in the St. Antonius Hospital in Nieuwegein. All patients undergoing isolated CABG in July 2014 were included in this registry. There were no exclusion criteria. Baseline data, antiplatelet treatment and postoperative complications (with 30 days of follow-up) were registered in all patients. The study was conducted according to the principles of the Declaration of Helsinki and received approval from the local Human Research Ethics Committee. The local ethics committee provided a waiver for written informed consent, as the study was not associated with any risk.

## Results

### Survey

Between November 2014 and April 2015, 11 out of the16 Dutch centres in which cardiothoracic surgery is performed, including our own centre, responded to the questionnaire. The other centres are listed in the acknowledgements.

**Fig. 1a–c:**
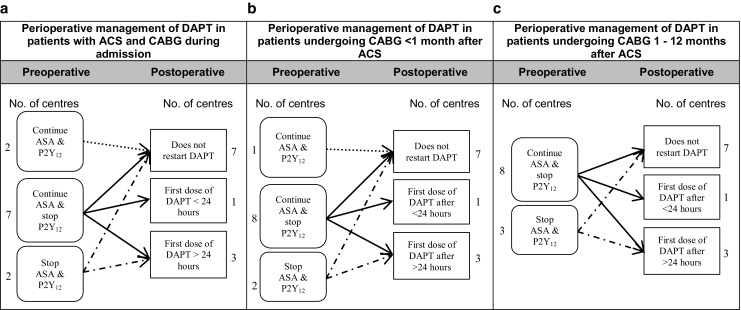
Perioperative management of DAPT in patients with ACS and CABG during the same admission, CABG < 1 month and CABG 1–12 months after ACS. *ACS* acute coronary syndrome, *ASA* acetylsalicylic acid, *CABG* coronary artery bypass grafting, *DAPT* dual antiplatelet therapy, *No* number, *P2Y*_*12*_ P2Y_12_ inhibitor

**Fig. 2a–c:**
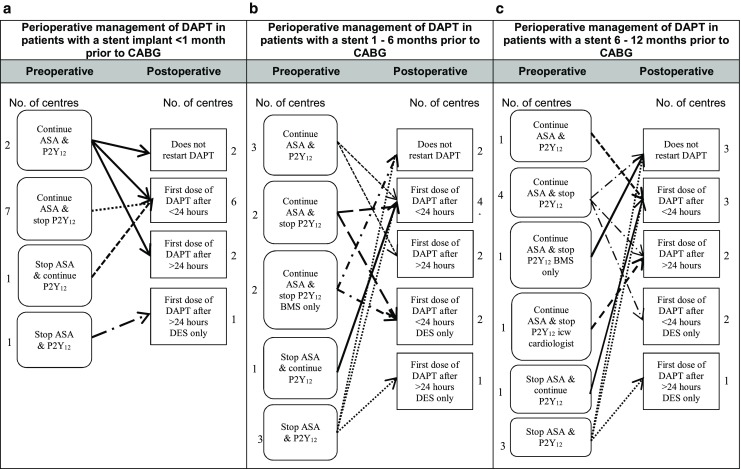
Perioperative management of DAPT in patients undergoing CABG less than 1 month, between 1–6 months and between 6–12 months after stent implantation. *ASA* acetylsalicylic acid, *BMS* bare metal stent, *CABG* coronary artery bypass grafting, *DAPT* dual antiplatelet therapy, *DES* drug-eluting stent, *ICW* in consultation with, *No* number, *P2Y*_*12*_ P2Y_12_ inhibitor

Six out of 11 centres answered that ASA monotherapy was routinely discontinued before CABG, while 5 centres always continued ASA monotherapy (Table 1). Fig. 1a to 1c show the perioperative management of antiplatelet treatment in patients with ACS undergoing CABG during the index admission, between the index admission and 1 month after the ACS or between 1 month and 12 months after the ACS. Preoperative management differed slightly between centres, but postoperative management was the same for the different groups. Fig. 2a to 2c show the perioperative management of antiplatelet treatment in patients undergoing CABG within 1 month, between 1 and 6 months and between 6 and 12 months after coronary stent implantation.

**Table 1:** Preoperative management of antiplatelet therapy

Number of centres following each strategy				
	ASA monotherapy (N)	DAPT Clopidogrel (N)	DAPT Prasugrel (N)	DAPT Ticagrelor (N)
Continue:	5	–	–	–
Discontinue:				
1 day	–	–	–	–
2 days	–	–	–	1
3 days	1	–	–	–
4 days	1	1	1	1
3–5 days	1	–	–	–
5 days	2	1	3	5
5–7 days	–	–	–	1
7 days	–	1	2	1
7–10 days	1	6	1	–

The preoperative management of patients on ASA monotherapy and patients on DAPT with clopidogrel, prasugrel or ticagrelor

*ASA* acetylsalicylic acid, *DAPT* dual antiplatelet therapy, *N* number of centres

In the 4 centres in which ASA treatment was discontinued before surgery, one centre discontinued ASA 5 days before surgery, a second centre 4 days, a third centre 3 days and the fourth centre 2 days prior to surgery. This discrepancy in the time of discontinuation of treatment also applied to the management of P2Y_12_ inhibitor use (Table 1).

### Registry

A total of 70 patients underwent isolated CABG in the St. Antonius Hospital in Nieuwegein in July 2014 and were included in this pilot study. Baseline data are presented in Table 2. Of these 70 patients, 41 were on ASA monotherapy, 28 used a P2Y_12_ inhibitor and 1 patient was on ASA plus acenocoumarol treatment before surgery. From the 28 patients using a P2Y_12_ inhibitor, 9 were on clopidogrel and 19 were on ticagrelor.

**Table 2:** Baseline characteristics

Characteristics	*N* = 70 *N* (%)
*Patient characteristics*	
Male	57 (81.4)
Age, mean, (SD), years	65.5 ± 10.1
Body mass index, mean, (SD)	28.0 ± 3.2
Current smoker	14 (20.3)
Ex-smoker (>6 weeks)	19 (27.5)
Family history for CAD	8 (12.1)
*Medical history*	
Hypertension	58 (82.9)
Diabetes mellitus	21 (30.0)
Dyslipidaemia	37 (52.9)
Angina pectoris month prior to surgery*	50 (71.4)
TIA/Stroke	6 (8.6)
COPD	6 (8.6)
Chronic kidney disease (eGFR MDRD4 < 60 ml/min)	3 (4.3)
Peripheral arterial disease	3 (4.3)
Heart failure (NYHA class III or IV)	9 (12.9)
ACS	39 (55.7)
– MI	34 (48.6)
Prior PCI	18 (26.1)
– PCI + Stent	11 (15.7)
Prior CABG	0 (0.0)
*Preoperative medication use*	
Oral nitrates	15 (21.4)
Beta-blockers	57 (81.4)
ACE inhibitor	38 (54.3)
AT-II-antagonists	17 (24.3)
Diuretics	18 (25.7)
Statins and other lipid-lowering drugs	67 (95.7)
Oral antidiabetics	16 (22.9)
Insulin	8 (11.4)
*Surgery*	
Coronary artery disease	
– One vessel	10 (14.3)
– Two vessel	9 (12.9)
– Three vessel	51 (72.9)
Timing	
– Elective/planned	66 (94.3)
– Urgent	2 (2.9)
– Emergency	2 (2.9)
EuroScore (SD)	3.2 ± 2.6

Data are presented as number and percentage unless otherwise indicated. Denominators to derive percentages are based on available data for each characteristic. *Any Canadian Cardiovascular Society class angina

*ACE* angiotensin-converting-enzyme, *ACS* acute coronary syndrome, *AT-II *Angiotensine-II, *CABG* coronary artery bypass grafting, *CAD* coronary artery disease, *COPD* chronic obstructive pulmonary disease. eGFR MDRD: estimated glomerular filtration rate according to the modification of diet in renal disease formula, *CVA* cerebral vascular accident, *TIA* transient ischaemic attack, *LMWH* low-molecular-weight heparin, *MI* myocardial infarction, *N* number of patients, *NYHA* New York Heart Association functional classification, *(N)OAC* (non-)vitamin K antagonist oral anticoagulant, *PCI* percutaneous coronary intervention, *SD* standard deviation

**Fig. 3:**
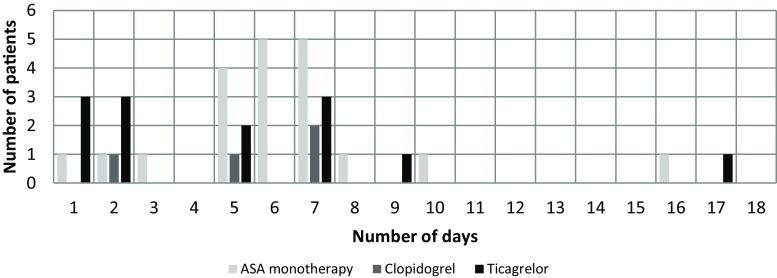
Number of days ASA was discontinued preoperatively in patients on ASA monotherapy and the number of days clopidogrel and ticagrelor were discontinued in patients on DAPT. *ASA* acetylsalicylic acid, *DAPT* dual antiplatelet therapy

Table 3 shows the preoperative management for patients treated with ASA monotherapy, for patients using clopidogrel as part of DAPT treatment and for patients using ticagrelor as part of DAPT treatment. Of the total of 70 patients, 2 were on clopidogrel due to intolerance for ASA. One of them continued to use clopidogrel. One patient was treated with triple therapy (ASA/clopidogrel/acenocoumarol) and the last patient was treated with clopidogrel and acenocoumarol. The patient on triple therapy continued the acenocoumarol and stopped ASA and clopidogrel. The patient on acenocoumarol plus clopidogrel treatment continued the acenocoumarol and stopped the clopidogrel. Fig. 3 shows the number of days that medication was discontinued preoperatively. In the group of patients who discontinued ticagrelor, 8 patients had experienced an ACS less than 1 month before surgery. In the group that continued ticagrelor, 5 patients had experienced an ACS within 1 month before surgery.

**Table 3:** Management of antiplatelet therapy in the pilot study

	ASA monotherapy	DAPT clopidogrel	DAPT ticagrelor
*Preoperative*			
Continued	21	1	6
Discontinued	20	4	13
Days discontinued, median (IQR)	6 (2)	6 (3.5)	5 (5.5)
*Postoperative*			
No restart	0	2	10
Restart	20	2	3
Days after CABG until restart, median (IQR)	1 (0)	2.5 (3)	1 (2)

Preoperative management of patients on ASA monotherapy and of clopidogrel and ticagrelor in patients on DAPT

*ASA* acetylsalicylic acid, *DAPT* dual antiplatelet therapy

After surgery, 68 out of 70 patients received ASA. Treatment was started the day after surgery in all patients. All these patients received a loading dose of 500 mg intravenously. The two patients who did not receive ASA postoperatively were both preoperatively treated with clopidogrel monotherapy due to ASA intolerance. Both these patients received their first doses of clopidogrel the day after surgery.

**Fig. 4a,b:**
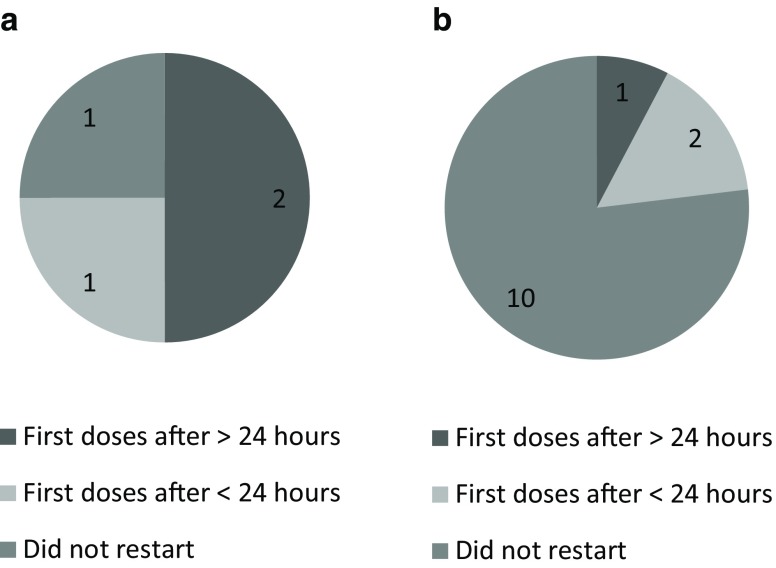
Postoperative management of clopidogrel and ticagrelor in patients preoperatively on DAPT. *DAPT* dual antiplatelet therapy

Fig. 4 shows the postoperative management of P2Y_12_ inhibitors. Patients who received a P2Y_12_ inhibitor after CABG did not receive a loading dose, but a regular maintenance dose. There was no apparent relationship between restarting DAPT postoperatively and a preoperative history of ACS or percutaneous coronary intervention (PCI) with stent implantation. The incidence of postoperative complications within 30 days was low. Myocardial infarction (MI), stroke and death were not observed, while Bleeding Academic Research Consortium (BARC) type 4 major bleeding occurred in 3 patients and 2 patients needed surgery for mediastinitis. Due to the small study population with subgroups and low incidence of postoperative complications, we decided not to analyse these postoperative complications in more detail.

## Discussion

The results from this national survey regarding the perioperative management of antiplatelet treatment in CABG patients show major variability across the different Dutch centres. This variability partly reflects the disparity in recommendations in the different international guidelines [1–4]. A survey regarding antithrombotic treatment in CABG patients conducted in 1989 also showed major differences in antithrombotic treatment between Dutch cardiothoracic centres [7]. Although some treatment strategies that were used at the time (coumarins and dipyridamole) have since been abandoned for routine use in CABG patients, the variability in treatment strategies persists.

### CABG in patients using ASA monotherapy

The management of patients with ASA monotherapy differs greatly between centres, both in continuing or discontinuing ASA before surgery and in the timing of discontinuation. Although it might be reasonable to stop ASA in patients with the highest bleeding risk [3, 4], the guidelines do not support discontinuation of ASA in the majority of patients, contrary to what appears to be the standard in many cardiothoracic centres who responded to this survey. The differences regarding the preoperative discontinuation of ASA in the guidelines and routine treatment in these centres might be caused by a lack of convincing evidence from randomised clinical trials. A recent meta-analysis including a total of 2399 patients showed that ASA exposure within 7 days before CABG, with or without concomitant surgery, resulted in a 44% reduction in the odds of MI [8]. However, it also resulted in a dose-dependent increase in blood loss, an increased volume of red cell transfusion and rate of surgical re-exploration without an effect on mortality.

In the largest randomised clinical trial to date regarding the preoperative use of ASA in CABG patients (n = 2100), [9] no differences were observed between patients using ASA or placebo with regard to any of the primary outcomes, death, MI, stroke, renal failure, pulmonary embolism and bowel infarction at 30 days after surgery (RR 0.94 95% CI 0.80–1.12). There were also no significant differences in the number of reoperations for bleeding or cardiac tamponade. Therefore, there is no clear benefit of ASA treatment before CABG.

The postoperative use of ASA in CABG patients is recommended in all guidelines as it has been proven to increase venous graft patency and reduces the occurrence of ischaemic events during follow-up in all patients regardless of revascularisation strategy [10–13].

### CABG in patients using DAPT

Our survey revealed that the discrepancies in treatment strategies between the different centres were even greater in patients who received DAPT. Centres also differed in postoperative antiplatelet management, but the majority of centres stop the P2Y_12_ inhibitor without restarting it after surgery. The timing of discontinuation of the P2Y_12_ inhibitor varied roughly from 4 to 10 days between centres. A recent study from Hansson et al. shows that it is safe to discontinue ticagrelor 3 days and clopidogrel 5 days prior to CABG [14]. Many guidelines mention the option of preoperative bridging therapy with small molecule GPIIb/IIIa inhibitors (i. e. eptifibatide or tirofiban) or cangrelor after discontinuation of P2Y_12_ inhibitors in patients with an increased risk for ischaemic events (e. g. with recently implanted drug-eluting stent (DES)), but there is still little clinical evidence for this strategy. None of the respondents mentioned the use of this strategy in their centre.

Not restarting the P2Y_12_ inhibitor after surgery is not supported by the guidelines, which recommend restarting DAPT after CABG as soon as it is considered safe and to continue DAPT for at least 12 months following ACS (class I, level A) [3, 15]. The ESC guidelines on revascularisation and non-ST-segment elevation ACS (NSTE-ACS) were updated after we received answers for our survey, but the 2011 ESC guideline on NSTE-ACS also stated that ticagrelor or clopidogrel should be considered to be (re-)started after CABG surgery as soon as considered safe (class IIa, level B) [16]. The recommendations from these guidelines are based on sub-analyses from three large randomised trials in ACS patients: the Clopidogrel in Unstable Angina to Prevent Recurrent Events (CURE) study [17], the TRial to Assess Improvement in Therapeutic Outcomes by Optimizing Platelet InhibitioN with Prasugrel–Thrombolysis In Myocardial Infarction (TRITON-TIMI 38) and the PLATelet inhibition and patient Outcomes (PLATO) study [18, 19]. These three trials all showed some benefit of continuation of DAPT after CABG in sub-analyses. However, the trials are underpowered and these post-hoc analyses have many limitations. The reason for the discontinuation of study treatment after surgery in a substantial number of patients is not reported in these trials, so there might be a selection bias. Furthermore, the percentage of patients who underwent CABG was relatively low (16.5% in CURE, 2.5% in TRITON-TIMI 38, and 6.8% in PLATO).

There is still much uncertainty as to how P2Y_12_ inhibitor treatment improves clinical outcome in this group and which patients should receive it at which particular moment. Outcomes might be improved due to an increase in vein graft patency with the use of a P2Y_12_ inhibitor, as vein graft occlusion occurs in up to 26% of grafts after 1 year in patients using ASA monotherapy [20]. Multiple studies investigating the routine use of P2Y_12_ inhibitors in CABG patients are currently recruiting patients, including the The Effect Of Ticagrelor On Saphenous Vein Graft Patency In Patients Undergoing Coronary Artery Bypass Grafting Surgery (POPular CABG) study (clinicaltrials.gov identifier NCT02352402) and the Study Comparing Ticagrelor With Aspirin for Prevention of Vascular Events in Patients Undergoing CABG (TiCAB) study (clinicaltrials.gov identifier NCT01755520). Data from these studies might help us better assess the benefits and risks of antiplatelet therapy in all patients undergoing CABG.

### CABG after PCI without prior ACS

For patients undergoing CABG after PCI without prior ACS it is recommended to continue DAPT for at least 1 month after implantation of a bare metal stent (BMS) (class I, level A) and at least 6 months after a new-generation DES (class I, level B )[4]. However, the guidelines do not specify which postoperative therapy is advised if the target vessel has been bypassed.

The guidelines offer different options for the timing of both preoperative discontinuation and postoperative re-initiation of P2Y_12_ inhibitor treatment. It should be considered to withhold clopidogrel and ticagrelor for 5 days and prasugrel for 7 days prior to surgery (class IIA, level C) [1–4]. Postoperatively, DAPT should be restarted within 24 h if it is deemed safe, with a loading dose of the P2Y_12_ inhibitor to optimise vein graft patency (Class IIA, level C) [3, 4]. The guideline from the American College of Chest Physicians specifies that when CABG is performed less than 6 weeks after BMS or less than 6 months after DES, DAPT should be continued during surgery to prevent stent thrombosis (Grade 2C) [2].

### Registry results

The results of our registry in the St. Antonius Hospital demonstrate that there are major differences even in a single centre. Generally, the P2Y_12_ inhibitor is discontinued for a shorter time period before surgery than is advised by the guidelines. The reasons for this could be that patients were considered to be at higher risk for ischaemic events.

### Limitations

Multiple limitations regarding the survey and registry merit mention. A questionnaire will only result in a general depiction of clinical practice, although we tried to include open questions to gather information regarding treatment of patients who did not fall into standard treatment protocols. However, treatment might actually differ from the answers provided by the responders as individual physicians might deviate from local protocols. Furthermore, only 69% of centres responded to our questionnaire.

The pilot study is limited due to its single-centre nature and the small population size. Another limitation for both studies is that the new ESC guideline regarding non-ST-segment-elevation myocardial infarction was published after the survey and the registry were conducted. Adherence to the guidelines might have increased since.

## Conclusion

Dutch cardiothoracic centres are not unified in their perioperative management of antiplatelet therapy in patients undergoing isolated CABG. The lack of evidence from randomised controlled trials could contribute to these differences between centres. More evidence from ongoing trials is essential to better evaluate the benefits and risks of antiplatelet therapy in CABG patients and strengthen the recommendations of the guidelines.

## Caption Electronic Supplementary Material

Survey antiplatelet treatment
