# Supplementary material for: Perioperative management of antiplatelet treatment in patients undergoing isolated coronary artery bypass grafting in Dutch cardiothoracic centres
Source: Neth Heart J. 2017 Jun 13;25(9):482–9. doi: 10.1007/s12471-017-1006-z (PMC5571594; doi:10.1007/s12471-017-1006-z)
Supplement: Supplementary file 1 — Survey antiplatelet treatment [file 12471_2017_1006_MOESM1_ESM.docx]

**Antiplatelet treatment in CABG patients**

**Definitions used**

ACS= acute coronary syndrome (ST-elevated myocardial infarction, non ST-elevated myocardial infarction or unstable angina pectoris

ASA = acetylsalicylic acid

BMS = Bare metal stent

DES = Drug eluting stent

P2Y_12_ inhibitor = antiplatelet agents binding to the P2Y_12_ protein (clopidogrel (Plavix®, Vatoud®, Iscover®, prasugrel (Efient®) or ticagrelor (Brillique®)).

PCI = Percutaneous coronary intervention

**Questionnaire**

Please answer the following questions about the strategy used in your department. We ask you to answer all questions as completely as possible. We would like to ask you to print out, fill out and sign the questionnaire and then fax, email or mail it back to us. If there are any unclarities concerning the content of the questions, you can always contact us.

**1. Does your hospital have a protocol regarding antiplatelet therapy in patients undergoing CABG?**

YES / NO*

If you have answered YES to this question, we would like you to attach a copy of the last version of the protocol to this questionnaire.

**Preoperative phase:**

Please specify below how patiens are treated on your ward in daily clinical practice in each situation. Circle the most appropriate answer and fill in the entry fields.

**2. The patient is treated with ASA monotherapy preoperatively.**

2 a. Do you discontinue the ASA? YES / NO*

2 b. If you discontinue the ASA, how long do you discontinue it preoperatively?

____________________________________________________________________________________________________________

Comments:

____________________________________________________________________________________________________________

**3. The patient uses ASA and a P2Y_12_ inhibitor after a PCI with a stent placement <1 month prior to CABG.**

3 a. Do you discontinue the ASA? YES / NO*

3 b. If you answered YES, how long do you discontinue it preoperatively?

______________________________________________________

3 c. Do you discontinue the P2Y_12_ inhibtior? YES / NO*

3 d. If you answered YES, how long do you discontinue it preoperatively (and does the duration of discontinuation vary based on the specific drug)?

____________________________________________________________________________________________________________

* Circle the correct answer.

3 e. Are there groups of patients in which you deviate from the treatment described under 3a to 3d (e.g. BMS or DES)?

____________________________________________________________________________________________________________

3 f. Comments:

____________________________________________________________________________________________________________

______________________________________________________

**4. The patient uses ASA and a P2Y_12_ inhibitor after a PCI with stent placement >1 month and <6 months prior to CABG.**

4 a. Do you discontinue ASA? YES / NO*

4 b. If you answered YES, how long do you discontinue it preoperatively?

______________________________________________________

4 c. Do you discontinue the P2Y_12_ inhibitor? YES / NO*

4 d. If you answered YES, how long do you discontinue the drug preoperatively (and does this duration vary based on the specific drug)?

_________________________________________________________________________________________________________

4 e. Are there groups of patients in which you deviate from this (e.g. BMS or DES)?

____________________________________________________________________________________________________________

4 f. Comments:

____________________________________________________________________________________________________________

______________________________________________________

* Circle the correct answer.

**5. The patient uses ASA and a P2Y_12_ inhibitor after a PCI with stent placement >6 months and <1 year prior to CABG.**

5 a. Do you discontinue ASA? YES / NO*

5 b. If you answered YES, how long do you discontinue it preoperatively?

_____________________________________________________

5 c. Do you discontinue the P2Y_12_ inhibitor? YES / NO*

5 d. If YES, how long do you discontinue it preoperatively (and does this duration vary based on the specific drug)?

____________________________________________________________________________________________________________

5 e. Are there groups of patients in which you deviate from the treatment described under 5a to 5d (e.g. BMS or DES)?

____________________________________________________________________________________________________________

5 f. Comments:

____________________________________________________________________________________________________________

______________________________________________________

**6. The patient is admitted for ACS and is treated with ASA and a P2Y_12_ inhibitor. He or she undergoes a CABG during the same admission (no stent).**

6 a. Do you discontinue the ASA? YES / NO*

6 b. If you answered YES, how long do you discontinue it preoperatively?

______________________________________________________

6 c. Do you discontinue the P2Y_12_ inhibitor? YES / NO*

6 d. If YES, how long do you discontinue it preoperatively (and does this duration vary based on the specific drug)?

____________________________________________________________________________________________________________

6 e. Comments:

____________________________________________________________________________________________________________

______________________________________________________

* Circle the correct answer.

**7. The patient is treated with ASA and a P2Y_12_ inhibitor preoperatively after an ACS <1 month prior to CABG (no stent).**

7 a. Do you discontinue the ASA? YES / NO*

7 b. If you answered YES, how long do you discontinue it preoperatively?

______________________________________________________

7 c. Do you discontinue the P2Y_12_ inhibitor? YES / NO*

7 d. If YES, how long do you discontinue it preoperatively (and does this duration vary based on the specific drug)?

____________________________________________________________________________________________________________

7 e. Comments:

____________________________________________________________________________________________________________

______________________________________________________

* Circle the right answer.

**8. The patient uses ASA and a P2Y_12_ inhibitor preoperatively after an ACS >1 month and <1 year prior to CABG (no stent).**

8 a. Do you discontinue the ASA? YES / NO*

8 b. If YES, how long do you discontinue it preoperatively (and does this duration vary based on the specific drug)?

______________________________________________________

8 c. Do you discontinue the P2Y_12_ inhibitor? YES / NO*

8 d. If YES, how long do you discontinue it preoperatively (and does this duration vary based on the specific drug)?

____________________________________________________________________________________________________________

8 e. Comments:

____________________________________________________________________________________________________________

______________________________________________________

* Circle the correct answer.

**Postoperative phase:**

The next questions concern the policies after the surgery. **Restarting means that the medicine is administered after the surgery. The medicine could have been continued or discontinued prior to the CABG.**

**9. The patient used ASA and a P2Y_12_ inhibitor preoperatively because of a PCI with stent <1 month prior to the CABG.**

9 a. Do you restart the P2Y_12_ inhibitor? YES / NO*

9 b. If you answered YES, after how long do you restart it? ____________________________________________________________________________________________________________

9 c. Are there groups of patients in which you deviate from the treatment described under 9a and 9b (e.g. BMS or DES)? ____________________________________________________________________________________________________________

9 d. Comments:

____________________________________________________________________________________________________________

**10. The patient was treated with ASA and a P2Y_12_ inhibitor preoperatively after a PCI with stent >1 month and <6 months prior to the CABG.**

10 a. Do you restart the P2Y_12_ inhibitor? YES / NO*

10 b. If you answered YES, after how long do you restart it? ____________________________________________________________________________________________________________

10 c. Are there groups of patients in which you deviate from the treatment described under 10a and 10b (e.g. BMS or DES)?

____________________________________________________________________________________________________________

10 d. Comments:

____________________________________________________________________________________________________________

**11. The patient was treated with ASA and a P2Y_12_ inhibitor preoperatively after a PCI with stent >6 months and <1 year prior to the CABG.**

11 a. Do you restart the P2Y_12_ inhibitor? YES / NO*

11 b. If you answered YES, after how long do you restart it? ____________________________________________________________________________________________________________

* Circle the correct answer.

11 c. Are there groups of patients in which you deviate from the treatment described under 11a and 11b (e.g. BMS or DES)?

____________________________________________________________________________________________________________

11 d. Comments:

____________________________________________________________________________________________________________

**12. The patient was admitted for an ACS and was treated with ASA and a P2Y_12_ inhibitor preoperatively. During the same admission he undergoes a CABG.**

12 a. Do you restart the P2Y_12_ inhibitor? YES / NO*

12 b. If you answered YES, after how long do you restart it? ____________________________________________________________________________________________________________

12 c. Comments:

____________________________________________________________________________________________________________

**13. The patient was treated with ASA and a P2Y_12_ inhibitor preoperatively for an ACS <1 month prior to surgery (no stent).**

13 a. Do you restart the P2Y_12_ inhibitor? YES / NO*

13 b. If you answered YES, after how long do you restart it? ____________________________________________________________________________________________________________

13 c. Comments:

____________________________________________________________________________________________________________

**14. The patient was treated with ASA and a P2Y_12_ inhibitor preoperatively due to an ACS > 1 month and <1 year prior to surgery.**

14 a. Do you restart the P2Y_12_ inhibitor? YES / NO*

14 b. If you answered YES, after how long do you restart it? ____________________________________________________________________________________________________________

14 c. Comments:

____________________________________________________________________________________________________________

* Circle the correct answer.
